# Supplementary material for: Tumor microbiome diversity influences papillary thyroid cancer invasion
Source: Commun Biol. 2022 Aug 24;5:864. doi: 10.1038/s42003-022-03814-x (PMC9402670; doi:10.1038/s42003-022-03814-x)
Supplement: Supplementary file 2 — Supplementary Information [file 42003_2022_3814_MOESM2_ESM.pdf]

Supplementary Material

Supplementary Table S1 Clinical Characteristics of PTC cohort Patients

Patients Characteristics	Total number (n=80)	Percent (%)
**Gender**		
Male	19	23.75
Female	61	76.25
**Age, media (range), years**	43 (11-76)	
**T Stage***		
T1	35	43.75
T2	29	36.25
T3	10	12.50
T4	6	7.50
**Lymph node-metastasis (N *)**		
Yes	57	71.25
No	23	28.75
**Metastasis (M *)**		
Yes	2	2.50
No	78	98.50
**Subtypes of PTC**		
Classical PTC (CPTC)	66	82.50
Follicular PTC(FPTC)	14	17.50

*: The patients were considered for the T stage and lymphatic metastasis status (N), which is included in the TNM stages **(T** indicates the size of the main (primary) tumor and whether it has grown into nearby areas; **N** describes the extent of spread to nearby (regional) lymph nodes; **M** indicates whether the cancer has spread (metastasized) to other organs of the body**). T4**: The tumor is any size and has grown extensively beyond the thyroid gland into nearby tissues, no matter with the age.

Supplementary Table S2 Clinical Characteristics of PTC Patients with different T stages

Patients Characteristics	T1_2 (%)	T3_4 (%)	*P**	OR	95% CI
**Gender**			0.65	0.62	0.18-2.07
Male	14 (21.9)	5 (31.3)			
Female	50 (78.1)	11 (68.8)			
**Age, years**			0.22	2.01	0.65-6.20
≤43	35 (54.7)	6 (37.5)			
>43	29 (45.3)	10 (62.5)			
**Pathological subtype**			0.34	0.26	0.03-2.17
CPTC	51 (79.7)	15 (93.8)			
FPTC	13 (20.3)	1 (6.3)			
**Lymph node-metastasis**			0.39	0.60	0.19-1.91
Yes	47 (73.4)	10 (62.5)			
No	17 (26.6)	6 (37.5)			

*: calculated by chi-square.

Supplementary Table S3 Clinical Characteristics of the validation set PTC cohort patients

**Patients Characteristics**	**Total number (n=12)**	**Percent (%)**
**Gender**		
Male	3	25
Female	9	75
**Age, median (range), years**	40 (25-60)	
**T Stage**		
T1	11	91.67
T2	1	8.33
T3	NA*	NA
T4	NA	NA
**Lymph node-metastasis (N *)**		
Yes	7	58.33
No	5	41.67
**Metastasis (M *)**		
Yes	0	0
No	12	100%
**Subtypes of PTC**		
Classical PTC (CPTC)	12	100%
Follicular PTC(FPTC)	0	0

*data not available.

Supplementary Table S4 Clinical Characteristics of PTC Patients with validation set

Patients Characteristics	TT1_2 (%)	T3_4 (%)	*P**	OR	95% CI
**Gender**			0.72	1.36	0.25-7.32
Male	3 (25)	5 (31.3)			
Female	9 (75)	11 (68.8)			
**Age, years**			0.05	0.20	0.04-1.044
≤43	9 (75)	6 (37.5)			
>43	3 (25)	10 (62.5)			
**Pathological subtype**			0.39	0.94	0.83-1.06
CPTC	12 (100)	15 (93.8)			
FPTC	0 (0)	1 (6.3)			
**Lymph node-metastasis**			0.82	0.84	0.18-3.88
Yes	7 (58.3)	10 (62.5)			
No	5 (41.7)	6 (37.5)			

*: calculated by chi-square.

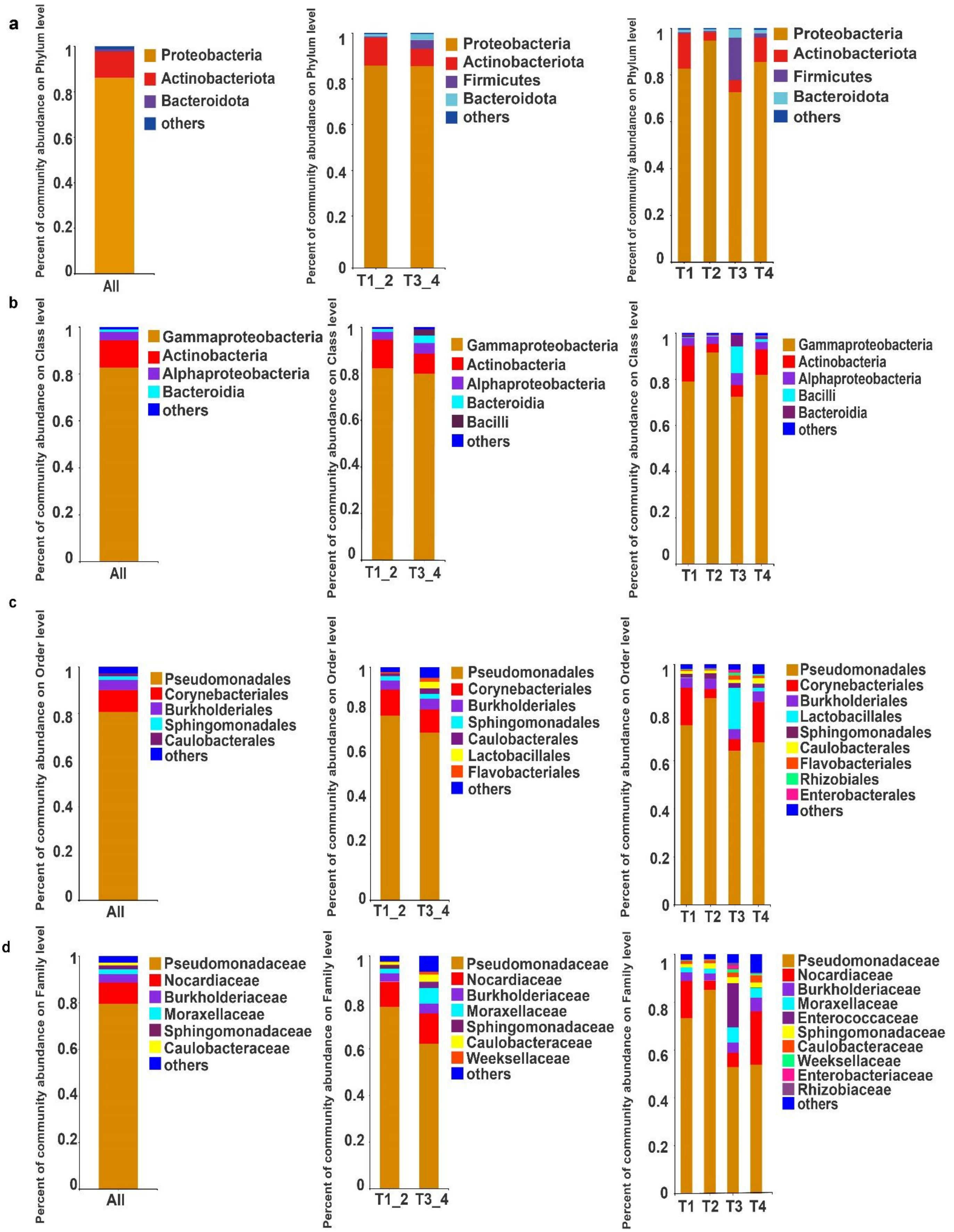

Supplementary Fig. S1. Tumor microbial communities at the different taxonomic levels significantly differ between clinical stages.

a) Bar plots of the phyla in patients with PTC in all patients, in patients with PTC in groups T1_2 and T3_4, in patients with PTC of clinical stages T1, T2, T3, and T4. **b)** Bar plots of the classes in patients with PTC in all patients, in patients with PTC in groups T1_2 and T3_4, and in patients with PTC of clinical stages T1, T2, T3, and T4. **c)** Bar plots of the orders in patients with PTC in all patients, in

patients with PTC in groups T1_2 and T3_4, and in patients with PTC in clinical stages T1, T2, T3, and T4. **d)** Bar plots of the families in patients with PTC in all patients, in patients with PTC in groups T1_2 and T3_4, and in patients with PTC of clinical stages T1, T2, T3, and T4.

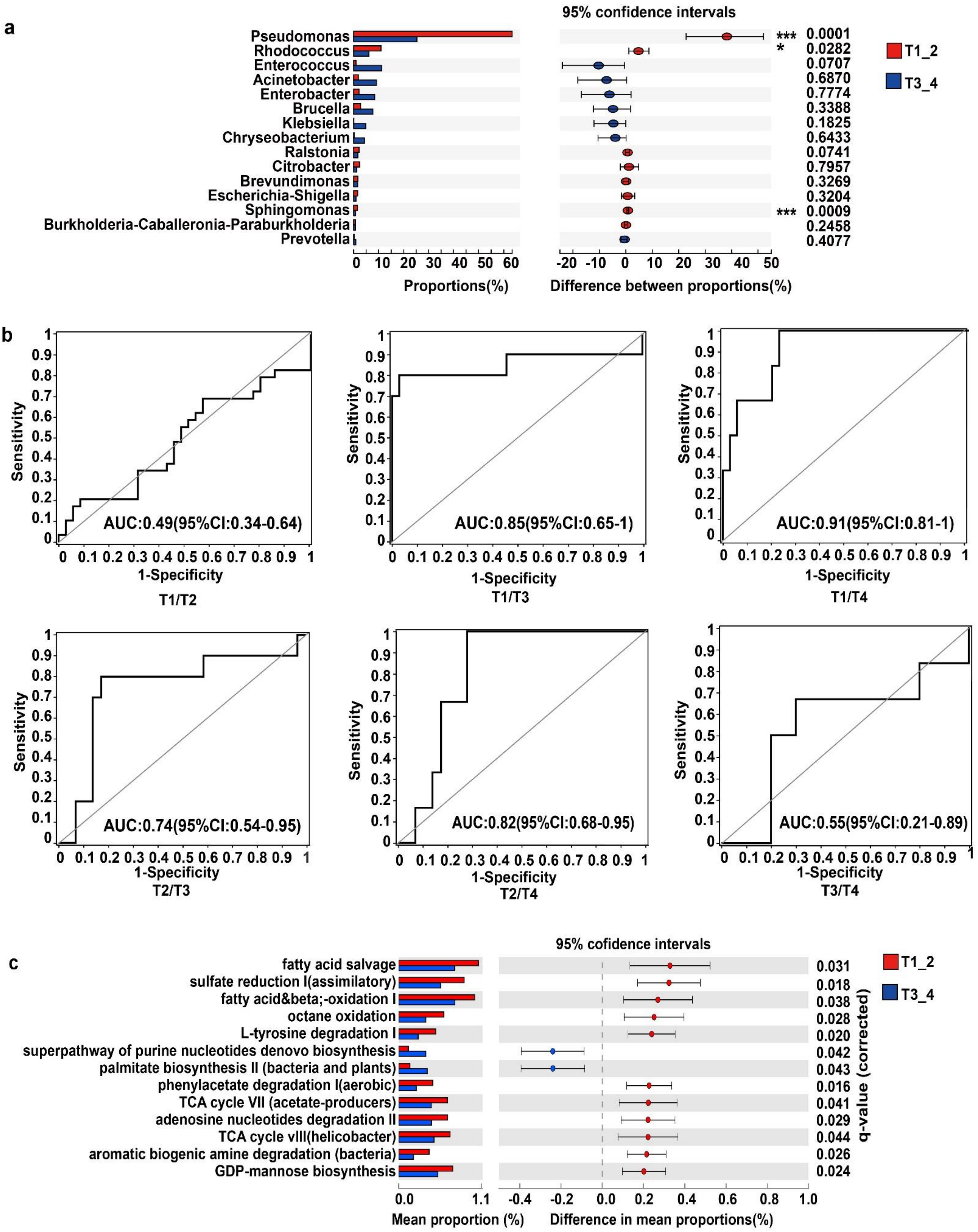

Supplementary Fig. S2. Tumor microbiome communities significantly differ among clinical stages.

a) Microbiome alterations at the genus level in patients with different PTC tumor invasion status. The differences between groups were analyzed by two-tailed Wilcoxon rank-sum tests, and the 95% CI was calculated using the bootstrap method.

b) ROC analysis of the relative abundances of taxa as a predictor of clinical T stage status. The top eight differential bacteria (genus) identified were tested between every two clinical T stages (p=0.4786 (T1/T2), p=0.0003 (T1/T3), p=0.0002 (T1/T4), p=0.018 (T2/T3), p=0.0070 (T2/T4), p=0.6436 (T3/T4)).

c) Functionally predicted MetaCyc pathways differing in proportions in patients with T1_2 and T3_4. The bar plot shows mean proportions of differential MetaCyc pathways predicted using PICRUSt2. The difference in proportions between the groups is shown with 95% confidence intervals. Only significant differences (p-value < 0.05; Welch's t test, FDR adjusted) are shown and composition.

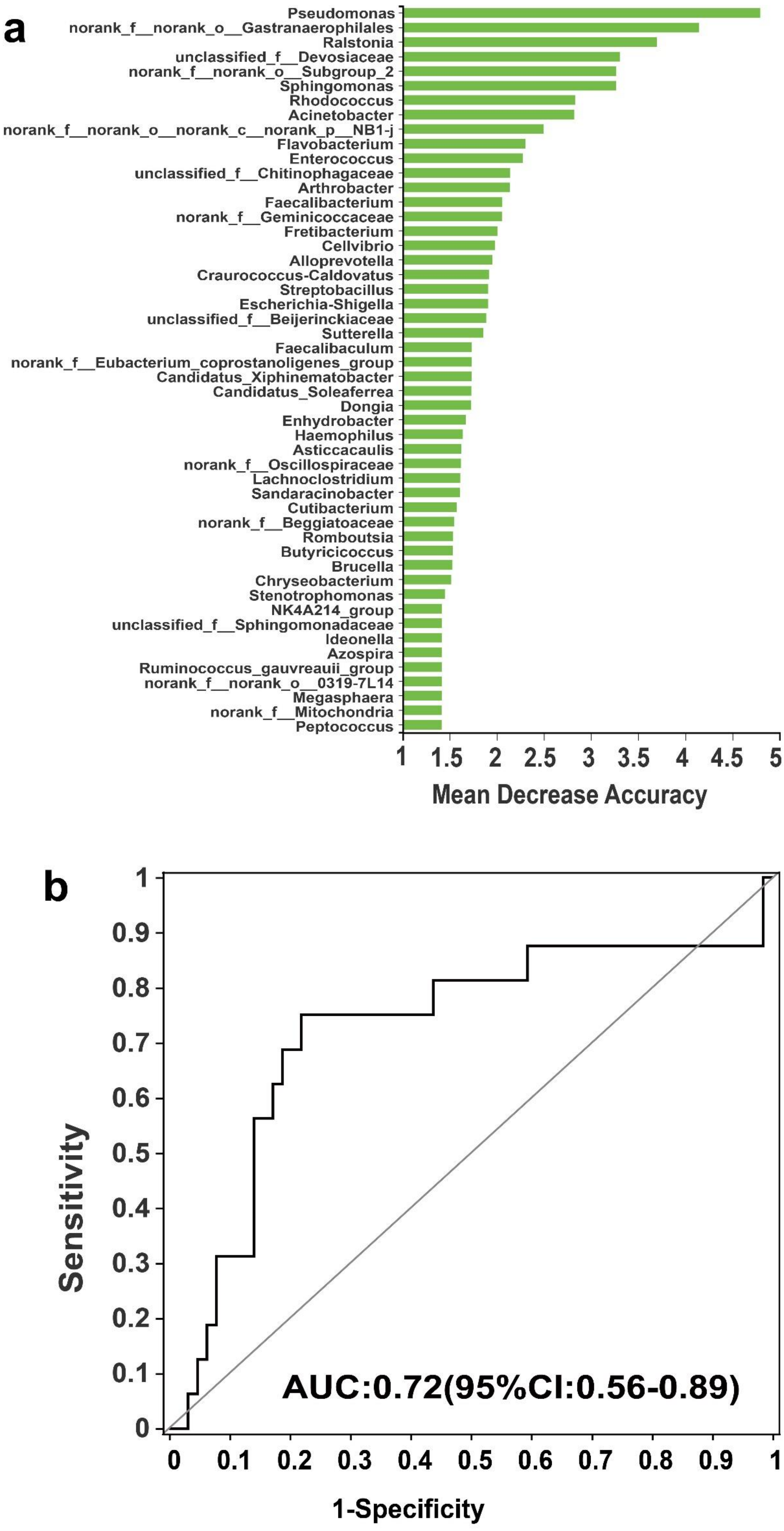
a
Pseudomonas
norank_f__norank_o__Gastranaerophilales
Ralstonia
unclassified_f__Devosiaceae
norank_f__norank_o__Subgroup_2
Sphingomonas
Rhodococcus
Acinetobacter
norank_f__norank_o__norank_c__norank_p__NB1-j
Flavobacterium
Enterococcus
unclassified_f__Chitinophagaceae
Arthrobacter
Faecalibacterium
norank_f__Geminicoccaceae
Fretibacterium
Cellvibrio
Alloprevotella
Craurococcus-Caldovatus
Streptobacillus
Escherichia-Shigella
unclassified_f__Beijerinckiaceae
Sutterella
Faecalibaculum
norank_f__Eubacterium_coprostanoligenes_group
Candidatus_Xiphinematobacter
Candidatus_Soleaferrea
Dongia
Enhydrobacter
Haemophilus
Asticcacaulis
norank_f__Oscillospiraceae
Lachnoclostridium
Sandaracinobacter
Cutibacterium
norank_f__Beggiatoaceae
Romboutsia
Butyricicoccus
Brucella
Chryseobacterium
Stenotrophomonas
NK4A214_group
unclassified_f__Sphingomonadaceae
Ideonella
Azospira
Ruminococcus_gauvreauii_group
norank_f__norank_o__0319-7L14
Megasphaera
norank_f__Mitochondria
Peptococcus
1 1.5 2 2.5 3 3.5 4 4.5 5
Mean Decrease Accuracy
b
Sensitivity
0 0.1 0.2 0.3 0.4 0.5 0.6 0.7 0.8 0.9 1
AUC:0.72(95%CI:0.56-0.89)
1-Specificity

Supplementary Fig. S3. Microbiome associations between microbiome communities from patients with T1_2 and T3_4 PTC

a) Order of important genera in discriminating T stages by random forest using cross validation. The top ten genera of bacteria were filtered when the average error rate was the lowest (the top ten genera of bacteria were filtered when the average error rate was the lowest [error rate=0.216]).

b) Receiver operating characteristic (ROC) curve of the relative abundance of bacterial taxa as a predictor of clinical T stage status (p=0.0002). The top ten bacterial genera were used and filtered using the random forest model.

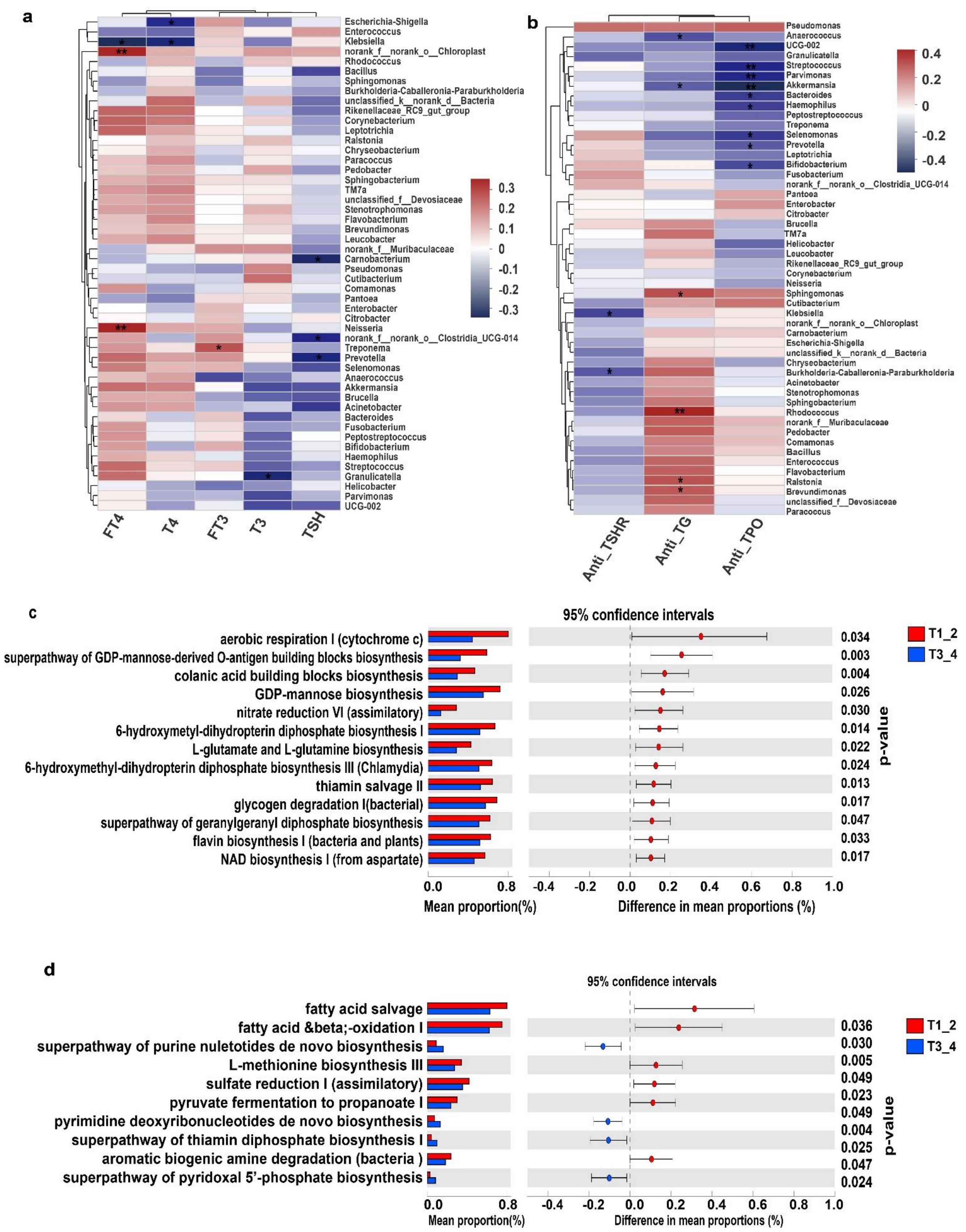

Supplementary Fig. S4. Heatmap of Spearman’s correlation analysis

between the general PTC intratumor microbiome and clinical factors.
a) Heatmap of Spearman’s correlation analysis between the general bacterial and thyroid-related hormones. *p<0.05 and **p<0.01. Heatmap color keys indicate spearman correlation coefficient.

b) Heatmap of Spearman’s correlation analysis between the general bacterial and thyroid diseases (AITD)-related antibodies. *p<0.05 and **p<0.01. Heatmap color keys indicate spearman correlation coefficient.

c) and **d)** Functionally predicted MetaCyc pathways differing in proportions in patients with T1_2 and T3_4 with special microbes correlated with **c)** thyroid hormones and **d)** autoimmune thyroid disease-related antibodies. The bar plot shows mean proportions of differential MetaCyc pathways predicted using PICRUSt2. The difference in proportions between the groups is shown with 95% confidence intervals. Only significant differences (p-value < 0.05; Welch’s t test, FDR adjusted) are shown and composition.

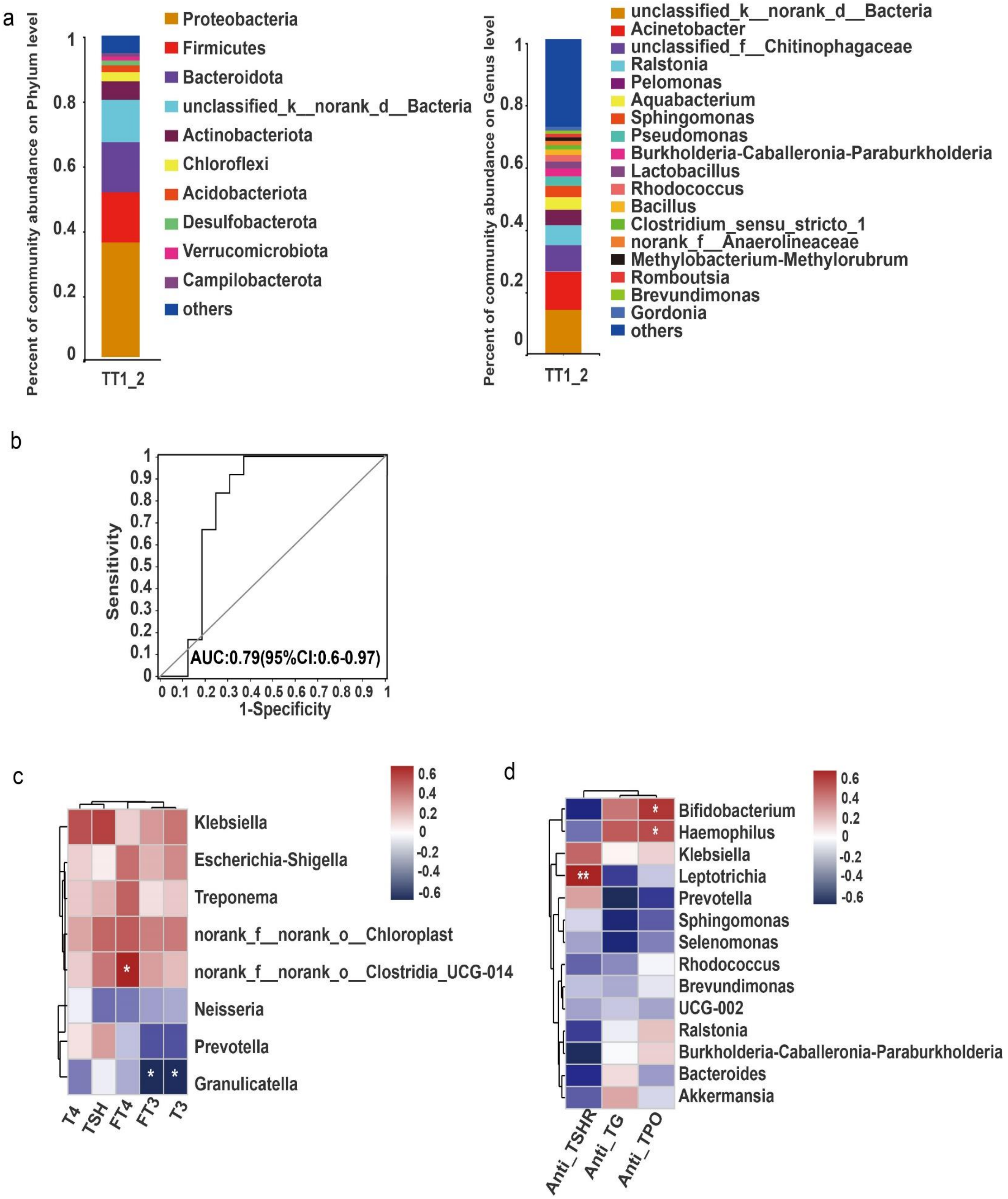

Supplementary Fig. S5. Changes in the intratumor microbiomes of patients with papillary thyroid carcinoma (PTC) in the validation set.

a) Tumor microbial communities at the different taxonomic levels (including phyla and genus). The validation set of patients with T1 or T2 PTC were assigned as group TT1_2.

b) ROC analysis of the relative abundances of taxa as a predictor of clinical T stage status in another set of samples (p=0.0057).

The validation set of patients with T1 or T2 PTC was assigned as group TT1_2.

c) Heatmap of Spearman's correlation analysis between the specific bacterial and thyroid-related hormones. *p<0.05 and **p<0.01. Heatmap color keys indicate spearman correlation coefficient.

b) Heatmap of Spearman's correlation analysis between the specific bacterial and thyroid diseases (AITD)-related antibodies. *p<0.05 and **p<0.01. Heatmap color keys indicate spearman correlation coefficient.

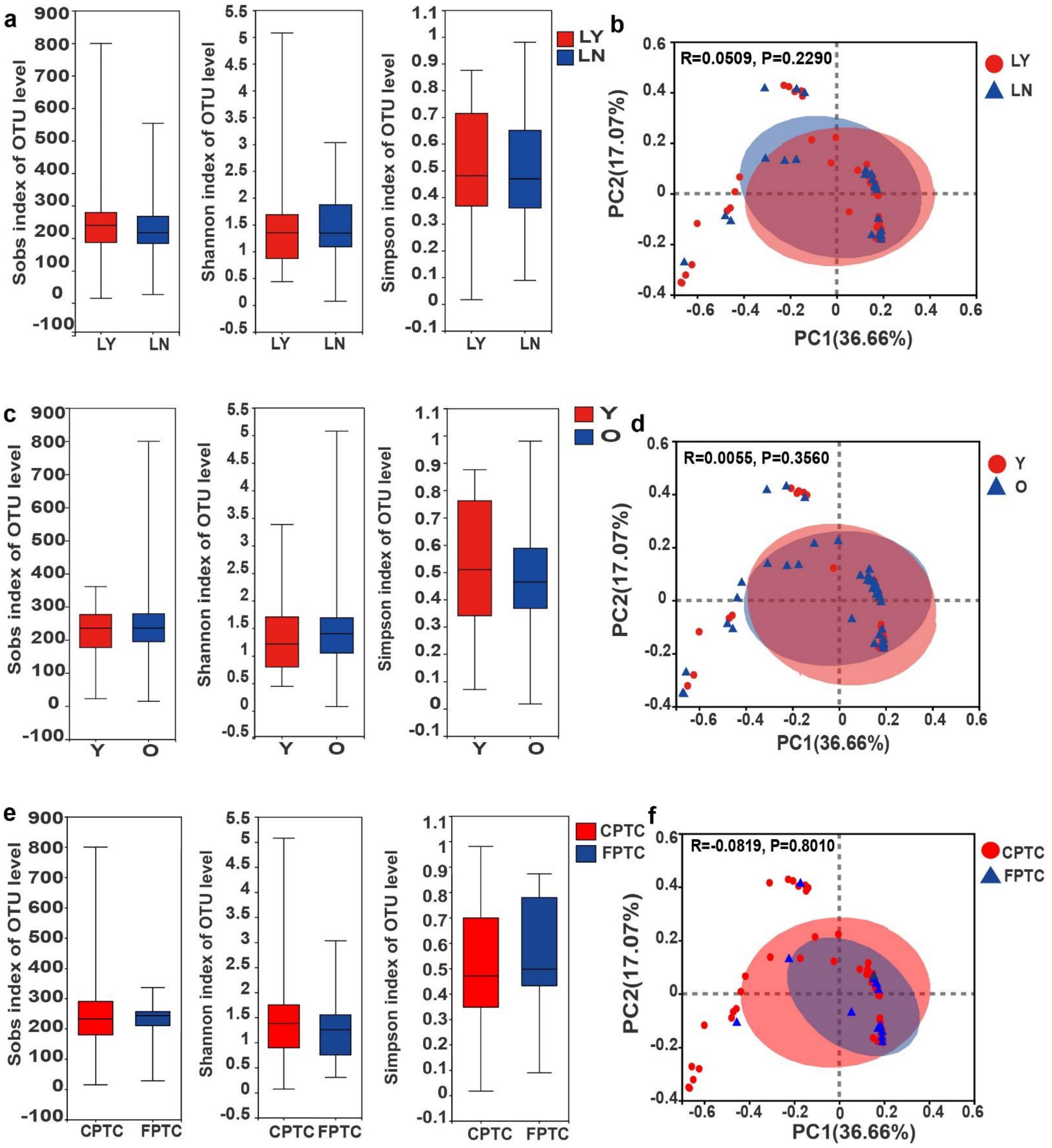

Supplementary Fig. S6. Changes in the intratumor microbiome in patients with PTC. a) α-Diversity at the OTU level (estimated using Sobs, Shannon, and Simpson indices) in patients of different ages with PTC. *p<0.05, **p<0.01. Error bars indicate SD. **b)** PCoA score plots based on Bray–Curtis distance at the OTU level in patients with PTC with PTC. Patients with PTC aged < 43 years were designated as group Y, and those aged ≥ 43 years were designated as group O. **c)** α-Diversity at the OTU level (estimated using Sobs, Shannon, and Simpson indices) in patients with PTC and lymphatic metastasis status (*p<0.05 and **p<0.01). Error bars indicate SD. **d**) PCoA score plots based on Bray–Curtis distance at the OTU level in patients with PTC and lymphatic metastasis status. Patients with PTC and lymphatic metastasis were designated as group LY, and those without lymphatic metastasis status were designated as group LN. Wilcoxon rank-sum test and ANOSIM

were used for analysis of intragroup difference of α-diversity and PCoA analysis, respectively. **e)** α-Diversity at the OTU level (estimated using Sobs, Shannon, and Simpson indices) in patients with PTC and subtypes (CPTC and FPTC) (*$p<0.05$ and **$p<0.01$). Error bars indicate SD. **f)** PCoA score plots based on the Bray–Curtis distance at the OTU level in patients with PTC and subtypes (CPTC and FPTC). Patients with CPTC were designated as the CPTC group, and those without FPTC were designated as the FPTC group. Wilcoxon rank-sum test and ANOSIM were used for analysis of intragroup difference of α-diversity and PCoA analysis, respectively.
