# Supplementary material for: Tumor microbiome diversity influences papillary thyroid cancer invasion
Source: Commun Biol. 2022 Aug 24;5:864. doi: 10.1038/s42003-022-03814-x (PMC9402670; doi:10.1038/s42003-022-03814-x)
Supplement: Supplementary file 3 — Description of Additional Supplementary Files [file 42003_2022_3814_MOESM3_ESM.pdf]

Description of Additional Supplementary Files

File name: Supplementary Data 1
Description: Source data underlying Fig 2a

File name: Supplementary Data 2
Description: Source data underlying Fig 2b

File name: Supplementary Data 3
Description: Source data underlying Fig 2c

File name: Supplementary Data 4
Description: Source date underlying Fig 2f

File name: Supplementary Data 5
Description: Source data underlying Fig 2h

File name: Supplementary Data 6
Description: Source data underlying Fig 3a

File name: Supplementary Data 7
Description: Source data underlying Fig 3b

File name: Supplementary Data 8
Description: Process data
